# Multiple Chromosome Fissions, Including That of the X Chromosome, in *Aulacocyclus tricuspis* Kaup (Coleoptera, Passalidae) from New Caledonia: Characterization of a Rare but Recurrent Pathway of Chromosome Evolution in Animals

**DOI:** 10.3390/genes14071487

**Published:** 2023-07-21

**Authors:** Bernard Dutrillaux, Anne-Marie Dutrillaux, Karen Salazar, Stéphane Boucher

**Affiliations:** 1 Institut de Systématique, Evolution, Biodiversité (ISYEB), Muséum National d’Histoire Naturelle, CNRS, Sorbonne Université, EPHE, Université des Antilles, 57 Rue Cuvier, CP 50 Entomologie, CEDEX 05, 75231 Paris, France; 2 Muséum National d’Histoire Naturelle, MECADEV UMR 7179 MNHN/CNRS, CP 50 Entomologie, CEDEX 05, 75231 Paris, France

**Keywords:** *Aulacocyclus*, Passalidae, New Caledonia, karyotype, autosome, X chromosome, fissions

## Abstract

The male karyotype of *Aulacocyclus tricuspis* Kaup 1868 (Coleoptera, Scarabaeoidea, Passalidae, Aulacocyclinae) from New Caledonia contains an exceptionally high number of chromosomes, almost all of which are acrocentric (53,X1X2Y). Unlike the karyotypes of other species of the pantropical family Passalidae, which are principally composed of metacentric chromosomes, this karyotype is derived by fissions involving almost all the autosomes after breakage in their centromere region. This presupposes the duplication of the centromeres. More surprising is the X chromosome fragmentation. The rarity of X chromosome fission during evolution may be explained by the deleterious effects of alterations to the mechanisms of gene dosage compensation (resulting from the over-expression of the unique X chromosome in male insects). Herein, we propose that its occurrence and persistence were facilitated by (1) the presence of amplified heterochromatin in the X chromosome of Passalidae ancestor, and (2) the capacity of heterochromatin to modulate the regulation of gene expression. In *A. tricuspis,* we suggest that the portion containing the X proper genes and either a gene-free heterochromatin fragment or a fragment containing a few genes insulated from the peculiar regulation of the X by surrounding heterochromatin were separated by fission. Finally, we show that similar karyotypes with multiple acrocentric autosomes and unusual sex chromosomes rarely occur in species of Coleoptera belonging to the families Vesperidae, Tenebrionidae, and Chrysomelidae. Unlike classical Robertsonian evolution by centric fusion, this pathway of chromosome evolution involving the centric fission of autosomes has rarely been documented in animals.

## 1. Introduction

Amongst the diverse chromosome rearrangements associated with speciation events, fissions are not the most commonly reported [1]. Large variations in chromosome numbers within and between species, genera, or families are more generally attributed to chromosome fusions, especially when the karyotype with the highest chromosome number is composed of acrocentric chromosomes. This process characterizes Robertsonian evolution. Unless evolution ineluctably tends to a reduction in chromosome numbers, the reverse process, i.e., a series of fissions, necessarily occurs. However, this can be difficult to demonstrate unless a series of derived chromosomes carrying different fragments of the same (ancestral) sequence are found in related taxa. For example, if two chromosomes with A and BCD, AB and CD, or ABC and D sequences are observed in three related species, it is likely that they all derive from the fission of the same unique ancestral ABCD chromosome [2]. To the best of our knowledge, this kind of evolutionary process, initially reconstructed after a chromosome banding analysis of Cercopithecinae (Primates) [3,4], has not been demonstrated in other taxa [5,6,7,8,9].

Geographical factors can also indicate the likelihood that the origin of a characteristic is derived rather than ancestral. In a family with a worldwide distribution, characteristics that are restricted to specimens from isolated populations are very likely to be derived rather than ancestral. We aim to establish whether this has occurred in Passalidae, a family of Coleoptera Scarabaeoidea. The presumed sister group of Passalidae is the extinct Passalopalpidae Boucher and Bai 2016 (−100 Ma), whereas their actual closely related taxa usually are thought to be in the Lucanidae, Trogidae and some other groups. The monophyletic and pantropical Passalidae are composed of two subfamilies: Passalinae Kaup, which is distributed worldwide, and Aulacocyclinae Kaup, which is restricted to Asia and Australasia (details in [10]).

In the archipelago of New Caledonia, about 1400 km east of Australia, the Passalid species, all belonging to the genus *Aulacocyclus*, display a series of noticeable particularities. Quite many species are present on such islands. All these species are endemic and may form a monophyletic group [10]. Their systematic composition and biogeography are exclusively related to Australia, and they are very distinct from their counterparts on the nearest eastern archipelago, Vanuatu (shortest distance: 200 km) [11]. These data inspired the present study of the New Caledonian species *A. tricuspis* Kaup 1868.

Almost all studies on Passalidae chromosomes have been performed on neotropical Passalinae, which are composed of two monophyletic tribes, Proculini Kaup and Passalini Kaup [10]. Passalini typically share 25,X/26,XX male and female karyotypes, whereas they may display large variations in diploid chromosome numbers (i.e., from 16 to 44 in Proculini) [12]. Such a large variation is not so common in Coleoptera, in which the 2 n = 20 number of chromosomes is widely spread [13]. This number is generally considered ancestral for all Coleoptera [13,14,15], and while it is the modal chromosome number of Lucanidae [16] and Trogidae [13], it is rare in Passalidae Passalinae, of which most studied species share 25,X male and 26,XX female karyotypes [12,13,15,17,18,19]. The large variations of chromosome numbers reported in Proculini are associated with various sex chromosome systems [12,17,20]. Thus, the composition of the primitive karyotype of Passalinae remains uncertain, but the modal 25,X male formula of Passalini is probably derived from the 20,XY ancestral karyotype via the loss of the Y chromosome and autosomal rearrangements (probably fissions). The large and variable size of the sub-metacentric X in Passalidae (about twice that of the average size of the X in Polyphagan beetles) is probably related to heterochromatin amplifications, as is suggested by its heterochromatic staining in male meiosis [21]. However, this has not been confirmed via negative C-banding, which is the usual method by which heterochromatin is characterized. The available cytogenetic data on Aulacocyclinae are scarce: only the karyotype of a single Australian species (*Aulacocyclus edentulus* (MacLeay, 1826)), composed of 30 chromosomes (30,XY in the male), almost all of which sub-metacentric, has been described [22]. We had the opportunity to study the karyotype of a second *Aulacocyclus* species: *A. tricuspis* from New Caledonia. Its unusually high number of chromosomes (53,X1X2Y), almost all of which are acrocentric, demonstrates that multiple chromosome fissions, including that of the X chromosome, have occurred. The causes and possible consequences of the fissions of the X chromosome are discussed below. Finally, a large survey of the literature on beetle chromosomes shows that the same chromosome particularities have been observed independently, though rarely, in a few other families, and this has enabled us to characterize a new pathway of chromosome evolution.

## 2. Material and Methods

Four male specimens of *A. tricuspis* were studied, collected in New Caledonia, Northern Province (Farino area and Mt Koniambo) in 2017, 2019 and 2022, under collection and export permits n° 60912-2001-2017 and 609011- 54/2022/DEPART/JJC. Voucher specimens were deposited at the Muséum National d’Histoire Naturelle, Paris (MNHN). Chromosome studies were performed on male testes. Unlike most other beetle families, the life span of Passalidae may exceed two years and their gametogenesis is prolonged by several months after emergence (personal observations). However, the best results are obtained with young specimens, which are identifiable by their brownish color (first weeks of life) and their non-worn mandibles. Cytogenetic techniques have been described in detail in [23]. The chromosome nomenclature used is derived from [24].

## 3. Results

The male karyotype of spermatogonia is composed of 53 chromosomes, of which 9 are metacentric/sub-metacentric and 44 acrocentric. The correct identification of sex chromosomes was made possible only after analysis of the other meiotic stages.

### 3.1. Spermatocytes I at Pachynema

**Figure 1:**
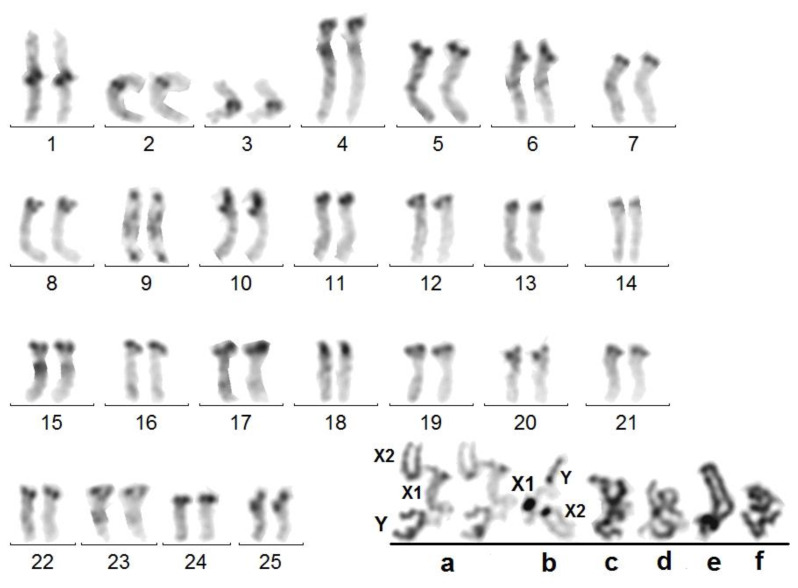
*A. tricuspis*.—For each number (1–25) and in a, sequentially Giemsa-stained and C-banded karyotype of a spermatocyte I at pachynema (centromere regions are similarly stained, except for sex chromosomes). All but n° 1–3 autosomal bivalents are acrocentric. (**a**–**f**): sex trivalents, (**a**): from this karyotype the C-banding makes centromeres more apparent. (**b**): C-banded sex trivalent from another cell. (**c**–**f**): Giemsa-stained sex trivalents from other cells, more compacted and heteropycnotic. When the sex chromosomes are distinct (**a**,**b**), they are not in synapsis, but chromosome X1 appears loosely associated with chromosomes X2 and Y. After silver staining (not shown), the NOR is located on the short arm of 2 bivalents (n° 10 and 23).

These spermatocytes display 25 bivalents (2 metacentric [n° 1 and 3], 1 sub-metacentric [n° 2] and 22 acrocentric [n° 4–25]), which correspond to 50 autosomes (Figure 1), and one sex trivalent often difficult to analyze (Figure 1c–f). One or two bivalents (n° 10 and 23) frequently exhibit an a-synaptic portion, which harbors the NOR (Nucleolus Organizer Region). When the three sex chromosomes are distinct, one is metacentric (X2) and two are submetacentric (X1 and Y) (Figure 1a,b). They remain in contact, but form neither synapsis nor chiasma.

### 3.2. Spermatocytes II

**Figure 2:**
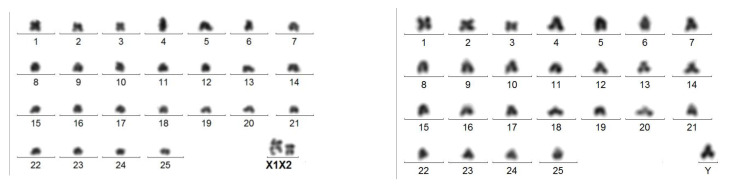
*A. tricuspis*.—Haploid karyotypes of a 27,X1X2 (**left**) and 26,Y (**right**) spermatocytes II with 3 submetacentric and 22 acrocentric autosomes.

**Figure 6:**
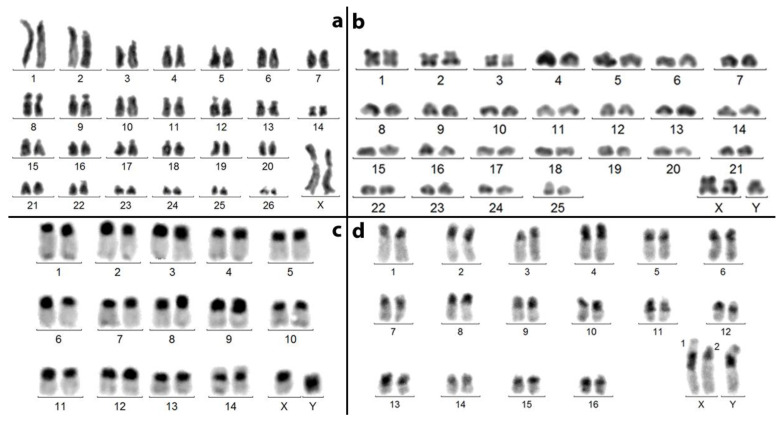
(**a**) 54,XX Giemsa-stained female karyotype of *V. xatarti*. Only pairs 14 and X are sub-metacentric, all other short arms are heterochromatic. (**b**) Giemsa-stained karyotype of *A. tricuspis*. (**c**) C-banded karyotype of *C. globicollis*. Only pair 14 is non-acrocentric. (**d**) C-banded karyotype of *B. mucronata* showing the same association of particularities as is *A. tricuspis*: high chromosome number, all or almost all acrocentric and multiple sex chromosomes.

As expected, spermatocytes II display two different karyotypes, with 27 and 26 chromosomes (Figure 2). Their comparison shows the common presence of 3 meta-/submetacentric and 22 acrocentric chromosomes which correspond to the 25 autosomes. One large sub-metacentric chromosome (the Y) complements the n = 26 formula, whereas one large sub-metacentric and one smaller metacentric chromosomes (X1 and X2) complement the n = 27 formula. Thus, the diploid male formula is 53,X1X2Y, which is confirmed by karyotypes of spermatogonia (see Figure 6).

### 3.3. Diakinesis

**Figure 3:**
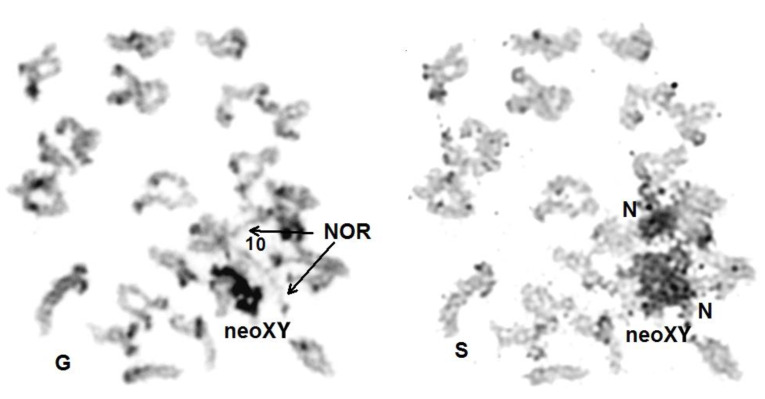
*A. tricuspis*.—Sequentially Giemsa (G) and silver (S) stained spermatocyte I at diakinesis. The NOR of bivalent 10 is expressed. The NOR carrier portions of each homolog chromosome 10 are not synapsed and uncoiled (arrows in G) and embedded in nucleolus (N) material in S. The heteropycnotic XXY trivalent (dark in G), here indicated as neoXY, is in contact with nucleolar material.

As is usual in other Passalidae species at this stage [21], the NORs of each carrier homolog are not synapsed, uncoiled and associated with abundant nucleolus material. The sex trivalent, completely heteropycnotic, is compacted and in contact with the nucleolar material (Figure 3).

### 3.4. Metaphase I

**Figure 4:**
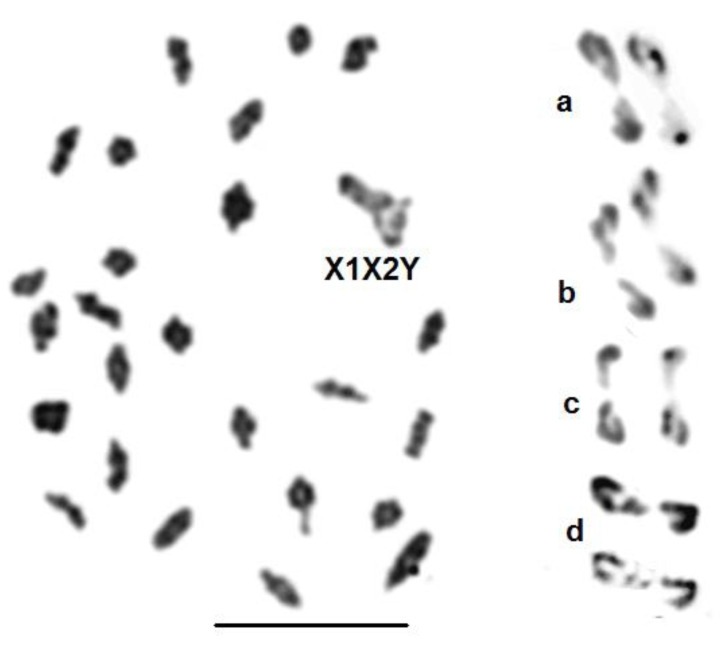
*A. tricuspis*.—Giemsa-stained metaphase I with compacted autosome bivalents (Bar scale 10 μm). (**a**–**d**) Sequentially Giemsa-stained (**left**) and C-banded (**right**) sex trivalents from other cells at late metaphase, showing the separation of the Y from the X1 and X2 chromosomes.

The sex trivalent is less compacted than the 25 autosomal bivalents (Figure 4). At metaphase/anaphase transition (Figure 4a–d) there is a 2:1 segregation of the sex chromosomes.

Finally, the analysis of the various meiotic stages indicates that, besides the 53,X1X2Y male karyotype, the female karyotype should be 54,X1X1X2X2. It also shows that, in spite of their large size, sex chromosomes, which become totally heteropycnotic at meiotic prophase, do not contain autosomal material.

## 4. Discussion

There is a consensus about the probable number of chromosomes (2 n = 20) composing the karyotype of the common ancestor of Polyphagan Coleoptera. This formula is found in about 25% of the 1136 diploid numbers in the data compiled in [13]. In this compilation, chromosome numbers above 20 are a majority (about 43%), but numbers above 35 are rare and largely represented by polyploidies observed in parthenogenetic species. No number above 50 is present. In our database (http://insect-cytogenetics.fr/ accessed on 17 January 2023), only *Vesperus xatarti* Mulsant, 1839 belonging to Vesperidae, a family related to Cerambycidae, has a chromosome formula of 2 n = 53/54 [25], similarly to *A. tricuspis*. As in *A. tricuspis*, the sex formula was not XY/XX (see below).

**Figure 5:**
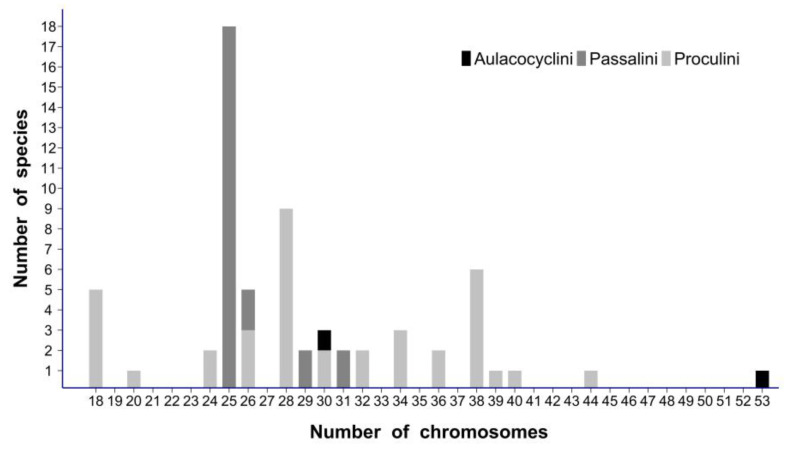
Histogram indicating the distribution of chromosome numbers in 3 tribes of Passalidae: Aulacocyclinae Aulacocyclini, Passalinae Passalini and Proculini.

To the best of our knowledge, 61/62 published chromosome numbers on Passalidae concern the subfamily Passalinae, and more precisely the two neotropical tribes Passalini and Proculini [12,13,17,18,19,20,25]. Passalini species have fairly stable karyotypes, generally composed of 25/26 chromosomes in males and females, respectively. Only 3/29 species differ, with 29 or 31 chromosomes in the male. Considering the 20,XY/20,XX karyotype of the presumed Scarabaeoidea ancestor, both the increase in the autosome numbers to 25/26 and the loss of the Y chromosome in the males seems to characterize the early chromosome evolution of Passalini [12]. On the other hand, the chromosomal evolution of Proculini looks complex. Their chromosome numbers range from 16 to 44 with a mode at 28, and the majority of them are above 28 (Figure 5). Thus, increases in chromosome numbers predominate, probably due to chromosome fissions, but fusions are likely to have also occurred [12]. There are no published data for other Passalinae, and those on Aulacocyclinae are limited to the 30,XY karyotype of the Australian *A. edentulus* (30,XY) [22]. This precludes from proposing any conclusion about the chromosomal evolution of this subfamily. However, the exceptional karyotype found in *A. tricuspis* warrants some comments.

### 4.1. Formation of Multiple Acrocentric Autosomes by Centromere Fission of Ancestral Metacentric Chromosomes: A Mechanism of Recent Karyotype Evolution Characteristic of New Caledonian Passalidae?

(1) The number of chromosomes of 53 in the male of *A. triscuspis* is among the highest known in Polyphagan beetles and the highest in Scarabaeoidea. In other Coleoptera, rare formulae above 40 were described in female karyotypes of Chrysomelidae and Curculionidae, two families in which parthenogenesis occurs. The origin of these high chromosome numbers is generally related to polyploidy, frequent in parthenotes, rather than to chromosome fissions. In rare instances, however, such as in the genus *Botanochara* (Chrysomelidae, Cassidinae) [26], both the increase in chromosome numbers up to 51 and the replacement of metacentric by acrocentric chromosomes are likely to indicate that chromosome fissions repeatedly occurred.

(2) Most karyotypes of Passalidae are composed of fewer than 30 chromosomes [12,17] (Figure 5) and when the morphology of chromosomes is indicated, almost all are sub-metacentric or metacentric. Thus, it is likely that multiple fissions originated the 44 acrocentric autosomes of the karyotype in *A. tricuspis*. This interpretation fits with the possible occurrence of multiple centromere fissions from a 30,XY karyotype, close or similar to that of *A. edentulus*, which is mainly composed of non-acrocentric chromosomes [22]. Indeed, amongst the karyotyped Passalidae, this Australian species of Aulacocyclinae is geographically the closest to New Caledonia.

(3) Considering the large variations of chromosome numbers in Passalidae, the number of 30 chromosomes in *A. edentulus* is not very far from the modal chromosome number of 25/26 in the subfamily Passalinae, but it may indicate that it was already derived by two chromosome fissions. More generally, the 53,X1X2Y karyotype of *A. tricuspis,* as well as the karyotypes with 36, 38 or 44 chromosomes of some Passalinae [12,17], suggest a general tendency towards an increase in the number of chromosomes in Passalidae. Thus, chromosome fissions were at work during the evolution of this family.

(4) Independently of their chromosome numbers, the published karyotypes of Passalidae are almost exclusively composed of non-acrocentric chromosomes. This strongly supports that presumed fissions in centromere regions would have been followed by pericentric inversions to form non-acrocentric chromosomes.

(5) Compared to the large variations of chromosome numbers in Passalidae, it is likely that relatively few chromosomal changes separated the ancestral karyotypes of the two sister group subfamilies, Passalinae and Aulacocyclinae (and therefore that of *A. edentulus*). The trend for increasing chromosome numbers in some Passalinae species but not in their congeneric species (see Table 2 in [12]) obviously indicates that many chromosome fissions were at work during their recent evolution, i.e., their speciation process. A recent accumulation of fissions is probably also at the origin of the karyotype of *A. tricuspis*. However, in the absence of chromosomal polymorphism in the four studied specimens of this species, data on other New Caledonian species are needed to determine whether the mechanism of fissions is widespread, even omnipresent, on the archipelago; in other words, whether this process characterized the first colonizing population of Passalids here.

(6) Finally, it is noteworthy that almost all chromosomes in *A. tricuspis* are acrocentric, contrary to other Passalidae. This may indicate the recent origin of the acrocentric chromosomes in said species, which have not yet undergone further rearrangements originating sub-metacentric/metacentric morphologies, as in neotropical species with a high number of chromosomes.

### 4.2. X Chromosome Fission in New Caledonian Passalidae: An Exceptional Event?

Besides the unusual high number of chromosomes, the presence of more than two sex chromosomes is another originality of the karyotype in *A. tricuspis*. In other beetles, the rare increases in sex chromosome numbers are most often due to the gain of the Y chromosome. However, the accidental formation of the XYY male sex formulae, as that of X0, is caused by the non-disjunction of the faulty Xyp system of Coleoptera, and not by chromosome fission [27]. Fissions involving the X chromosome during evolution of beetles, as in other animals, seem to be very unusual and not formerly demonstrated. This rarity is probably explained by the particular regulation of the X-linked genes. In mammals, compensating for the gene dosage imbalance between the XX female and XY male formulae, most genes of one X (the late replicating one) are down-regulated in female somatic cells through the cis-acting *Xist* RNA, which triggers gene silencing [28,29]. Any breakage of the X chromosome in XX females would separate a fragment from the Xist, which would escape to regulation, causing pathologies such as malformations and mental retardation [30]. Moreover, during the meiosis of XY males, sex chromosomes are inactivated, and the breakage of the X leads to sterility by the inopportune reactivation of normally silenced genes. Consequently, a strong selection exists against X chromosome rearrangements, which explains the high conservation of the X chromosome compared to autosomes during mammalian evolution. On the other hand, in insects, the compensation of the imbalance between XX females and XY or X0 males is principally achieved by the over-expression of the unique copy of the X-linked genes in somatic cells of males [31,32]. The mechanism involved is unknown, but the existence of a cis-acting message emitted by X-linked genes is likely, by analogy with the mammalian X regulation. In Polyphaga beetles, the X is amongst the smallest chromosomes, with a relative length of 5–6% (personal measurements). Its occasional enlargement is due to either heterochromatin amplification or X-autosome translocation [33]. Compared to the X chromosomes of most other Polyphaga, those of Passalidae are characterized by their large-sized X, more than twice larger than the average. However, both the frequent X0 formula of the males and the complete heterochromatization of the X chromosome during meiosis clearly discards the fact that an X-autosome translocation originates this size enlargement. Thus, the presence of gene free large heterochromatin components is the most likely hypothesis to explain the X chromosome size enlargement, in spite of its negative C-banding. A first possibility is that fissions could separate a non-coding portion from the transcriptionally active X proper part, without detrimental consequences. However, this does not explain the transmission during evolution, of a chromosome only composed of junk DNA. A second hypothesis is that the fission would have separated a fragment containing one or a few genes embedded in heterochromatin, thus insulated from the regulation of X proper genes in the ancestral chromosome, as suggested to explain the high occurrence of X-autosome translocations in beetles [33]. It is noteworthy that such X chromosome fissions could also constitute a mechanism originating B chromosomes by an amplification of centromeric heterochromatin of the X; a fission isolating the heterochromatic fragment, possibly with a few linked genes and imperfect centromere (B chromosome); and further faulty segregations leading to multiple B chromosomes under selection pressure.

### 4.3. X Chromosome Fission Associated with Multiple Centromere Fissions of Autosomes: A Rare, but Characteristic Pattern of Chromosome Evolution in Beetles

As seen above, strong increases in chromosome numbers in beetles are often a consequence of polyploidy, which recurrently occurs in parthenogenetic species, especially amongst the Polyphaga Chrysomelidae and Curculionidae. Indeed, these polyploid formulae, restricted to females, are of the 4N,XXXX type. On the other hand, multiple and variable sex chromosomes may be associated with a diploid number of autosomes, as in the Adephaga Cicindelidae, where formulae such as 22,X1X2X3Y/24,X1X1X2X2X3X3 are not rare [34]. In these karyotypes, the origin and composition of their sex chromosomes remain unknown, all or almost all autosomes remain non-acrocentric and their number is not increased.

Thus, the pattern of chromosome fission here described for *A. tricuspis* is particular but not limited to this species.

1. The 2 n = 53/54 karyotype of *V. xatarti* (Vesperidae) almost exclusively composed of acrocentric chromosomes (Figure 6a) [25] looks very similar to that of *A. tricuspis* (Figure 6b). The sex chromosome composition remains uncertain (only somatic cells were available) and the formula is either 53,X/54,XX or 53,neoX/54neoX neoX. The large phylogenetic distance between these two species indicates that this chromosome similarity is a convergence.
2. Amongst Chrysomelidae, a similar evolution probably occurred in three subfamilies. (A) In several species of the neotropical genus *Botanochara* (Cassidinae), increases in chromosome numbers from 2 n = 27 to 51 parallel the accumulation of acrocentric chromosomes and are associated with multiple sex chromosomes [26,35]. (B) In genus *Aulacophora* (Galerucinae) from India, increases in chromosome numbers from 2 n = 30 to 59 also parallel the formation of multiple sex chromosomes [36]. In genus *Isotes*, the 2 n male formula increases from 23,X in *Isotes multipunctata* to 37,X in *Isotes tetraspilota* [13]. (C) In genera *Pachybrachis* and *Cryptocephalus* (Cryptocephalinae), most of the species studied share either a 30,XY (16/35) or a 16,XY (7/35) male karyotype [37]. Our unpublished studies indicate that in the 30,XY karyotype of *Cryptocephalus globicollis* Suffrian, 1847 (Figure 6c, http://insect-cytogenetics.fr/ accessed on 17 January 2023), all autosomes but pair 14 are acrocentric, which is compatible with a derivation from the 16,XY karyotypes by repeated centric fissions of non-acrocentric chromosomes.
3. Amongst Tenebrionidae, some species of genus *Blaps*, such as *Blaps mucronata* Latreille, 1804 (Figure 6d, http://insect-cytogenetics.fr/ accessed on 17 January 2023), display a 2 n = 35,neoX1neoX2neoY male karyotype, in which all 32 autosomes are acrocentric (Figure 6d), whereas the number of autosomes is limited to 16 and 18 (non-acrocentric?) in other species, such as *Blaps judaeorum* and *Blaps cribosa* [38]. In this way, the same evolution by multiple centric fissions of autosomes may have occurred. There is also an increase in the number of sex chromosomes in this genus [13], but this is associated with an X-autosome translocation [33]. The 36,neoX1neoX2neoX3neoY/38,neoX1neoX1neoX2neoX2neoX3neoX3 male and female karyotypes of *Gnaptor spinnimanus* Pallas, 1781 [33] probably originated from the same process.

With the present example of *A. tricuspis*, we show that a quite similar evolution by multiple centric fissions of autosomes, often associated with either loss of the Y or rearrangements of sex chromosomes leading to the loss of the classical Xyp male sex formula, independently occurred in phylogenetically distant beetle taxa. The presence of intermediate stages, as in the genera *Blaps* and *Botanochara*, indicates that a progressive and coordinated phenomenon was at work. Duplications/amplifications of centromere DNA repeats as well as integration of telomere sequences may constitute predisposing conditions to form functional derivative chromosomes following fissions. However, the nature of these events remains as speculative as that of the presence of a probable selective pressure leading to transform n metacentric into 2 n acrocentric chromosomes, although ipso facto it increases the risk of meiotic aberrations and thus abnormal progeny. Finally, the fact that one example (*A. tricuspis*) of such chromosome evolution occurred in an island may indicate the role of recessive genetic determinisms.

On the whole, this study raises more questions than it provides answers, but it offers some possible tools to study the unanswered question of the mechanisms involved in chromosome number increases during evolution.

## Data Availability

Complementary data can be found in our database (http://insect-cytogenetics.fr/ accessed on 17 January 2023).
